# Personal exposure to air pollutants and immune system biomarkers in pregnant women

**DOI:** 10.1038/s41598-025-98712-7

**Published:** 2025-05-21

**Authors:** Anouk Marsal, Laurene Frau, Laurence Chaperot, Ines Amine, Sarah Lyon-Caen, Anne Boudier, Jean-Luc Jaffrezo, Rhabira Elazzouzi, Claire Philippat, Karine Supernant, Johanna Lepeule, Joane Quentin, Ryan Chartier, Sam Bayat, Remy Slama, Gaelle Uzu, Valérie Siroux

**Affiliations:** 1 University Grenoble Alpes, CNRS, INRAE, IRD, Grenoble INP, IGE, 38000 Grenoble, France; 2 University Grenoble Alpes, INSERM U1209, CNRS UMR 5309, Institut pour l’Avancée des Biosciences (IAB), Team of environmental epidemiology applied to development and respiratory health, 38000 Grenoble, France; 3 EFS, Recherche Et Développement, 38000 Grenoble, France; 4 Pediatric Department, CHU Grenoble Alpes, Grenoble, France; 5 Department of Pulmonology and Physiology, CHU Grenoble Alpes, Grenoble, France; 6 RTI International, Research Triangle Park, Durham, N.C. USA; 7 Inserm UA07 STROBE Laboratory, University Grenoble Alpes, Grenoble, France; 8 Agence de L’environnement Et de La Maîtrise de L’Energie, 20, Avenue du Grésillé, BP 90406, 49004 Angers Cedex 01, France; 9 University Grenoble Alpes, Inserm U1209, CNRS UMR 5309, Institut pour l’Avancée des Biosciences, Team of Epigenetics, Immunity, Metabolism, Cell Signaling & Cancer, 38000 Grenoble, France

**Keywords:** Immune system, Cytokines, Particulate matter, Oxidative potential, Nitrogen dioxide, Pregnancy, Risk factors, Epidemiology

## Abstract

The immune function is suspected to play an important role in the health effects of air pollution but it remains poorly investigated in pregnant women. One-week personal measurements of exposure to nitrogen dioxide (NO_2_), particulate matter with an aerodynamic diameter of ≤ 2.5 µm mass concentration (PM_2.5_) and PM_2.5_ oxidative potential (OP) were assessed in 270 pregnant women from the French cohort SEPAGES. PM filters were analyzed for PM_2.5_ OP using the dithiothreitol (DTT) and the ascorbic acid (AA) assays. From a blood sample withdrawn at the end of the exposure measurement week, levels of 29 cytokines and chemokines were measured at baseline and after T cell and dendritic cell activation with phytohemagglutinin (PHA) and resiquimod (R848), respectively. Associations between each air pollutant and each cytokine were assessed using adjusted linear regression models. An increase in NO_2_ exposure was associated with higher interleukin 10 (IL-10) and lower PHA-activated tumor necrosis factor (TNF). No association with PM_2.5_ concentration was observed, but increased exposure to PM ${\text{OP}}^{{{\text{AA}}}}$ was associated with lower baseline and R848-activated IL-8 and increased exposure to PM ${\text{OP}}^{\text{DTT}}$ was associated with higher PHA-activated IL-17A. Our study provides insights into the relationships between air pollution exposure and immune function among pregnant women.

## Introduction

Exposure to atmospheric pollutants, such as particulate matter (PM) and nitrogen dioxide (NO_2_), affects a number of human systems and organs, and contributes to a significant part of premature death worldwide^1^. Oxidative stress and the modulation of the immune system are two inter-related mechanisms through which air pollutants exert their deleterious effects on health^2–4^. Oxidative stress, by an excess of reactive oxygen species, can damage lipoproteins or lipids in membranes, leading to the formation of oxidation-specific epitopes recognized by pattern recognition receptors of the innate immune system^5^. This can enhance inflammatory mechanisms, thereby leading to a cyclic generation of oxidative stress and inflammation^6^.

Most of the research carried out on immune system has focused on the assessment of ambient particles, notably PM_10_ and PM_2.5_, and gases (NO_2_). Epidemiological studies showed that exposure to NO_2_ was associated with increased pro-inflammatory cytokines, such as IL-6 and TNF-α^7,8^. Previous studies highlighted that ambient exposure to fine particles was associated with an intensification of inflammatory responses and an increase in B lymphocytes^9,10^. The oxidative potential (OP) of PM, a metric developed to mimic oxidative stress generation in the interstitial lung fluid after PM inhalation, was assessed in very few epidemiological studies addressing its relationship with human immune system. OP^AA^, OP^DTT^ and OP^DCFH^ from ambient PM were found associated with elevated expression of pro-inflammatory biomarkers in lung epithelial cells^11,12^. To the best of our knowledge, four studies estimated associations between OP of PM and immune system biomarkers in humans and showed that OP of PM was positively associated with plasma IL-6^13^, blood IL-6 expression^14^ and IL-6 levels in nasal fluids^15^, while Steenhof et al.^16^ did not find any association with blood IL-6, nor with nasal IL-6 and IL-8. Overall, most studies tend to converge towards a pro-inflammatory effect of OP exposure, but they mostly relied on the expression of IL-6 and a relatively small sample size.

Pregnant women constitute a sensitive population to air pollution, primarily due to the modifications that occur in their immune systems during pregnancy, including a modification on cytokine production^17^. However, there is a notable lack of available data regarding effects of exposure to air pollution on the immune system in this particular population. Furthermore, it is especially relevant to delve into the mechanisms involved in the health effects of air pollution in pregnant women, as prenatal exposure to air pollution influences the development of child health and immune function^18–20^.

The aim of this study was to assess the associations between personal exposure to air pollutants (NO_2_, PM_2.5_ concentration and PM OP) during pregnancy and immune function measured both at the baseline state and after T cell and dendritic cell activation.

## Materials and methods

### Study population

**Fig. 1:**
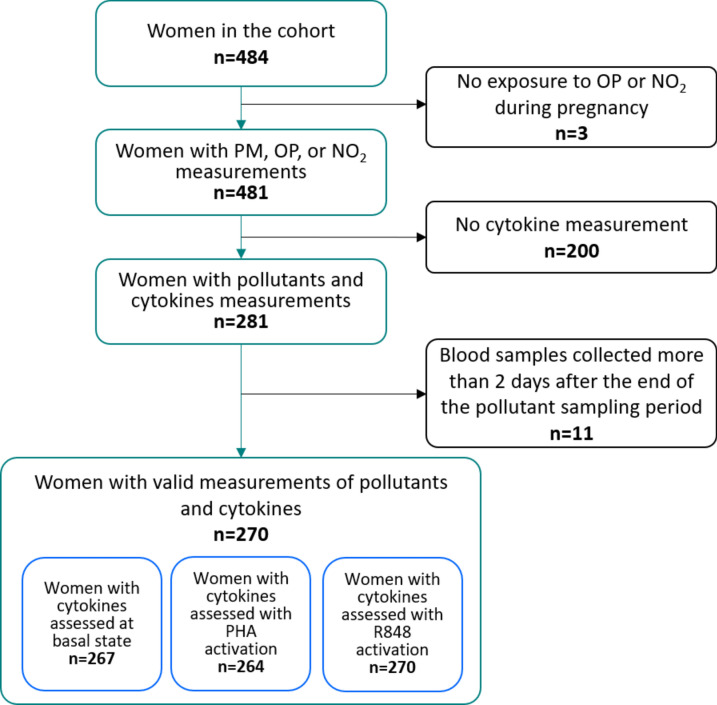
Flow chart for the selection of the study population. Abbreviations: PM_2.5_ particulate matter with diameter ≤ 2.5 μm; NO_2_ nitrogen dioxide; OP: oxidative potential; PHA phytohemagglutinin; R848 resiquimod.

This study is based on data from the SEPAGES French parent–child cohort. The population of the study lives within 80 km around the center of Grenoble in the Alpes, France. Briefly, 484 pregnant women, with pregnancy duration less than 19 weeks and with a singleton pregnancy, older than 18 years old and affiliated to the French national security system, were recruited between 2015 and 2017^21^. Their partner and children were also recruited. Sociodemographic and health data were collected using a combination of questionnaires, interviews, and clinical examinations during and after pregnancy. Exposure information were collected using personal samplers and immunological information were collected using blood samples. The present analysis is based on 270 mothers with exposure assessed to at least one of the measured air pollutants during pregnancy and immunological measurements at the end of the exposure assessment week (Fig. 1). To be included in the analysis, mothers had to have the blood samples collected within 2 days after the pollutant sampling period to assess the immune system’s status in relation to the exposure measurements. Participants signed an informed consent and the study protocol received approval from the French data privacy institution (Commission Nationale de l’Informatique et des Libertés, CNIL—n°914138) and the Comité de Protection des Personnes Sud-Est V (CPP—2013-A01491-44). All methods were performed in accordance with the relevant guidelines and regulations.

### Personal exposure assessment to air pollutants

Women in the SEPAGES cohort wore or kept nearby personal air samplers placed in a wearable backpack to measure their personal exposures to PM_2.5_ (MicroPEM™ active air sampler; RTI International, USA) and NO_2_ (Passam AG passive air sampler; Switzerland) for 7 (13%) to 8 (87%) consecutive days^22–24^. The measurement week took place during the second (81%) or the third trimester (19%) of pregnancy (median [min–max] gestational age = 19 weeks^14–36^).

NO_2_ concentration was measured using spectrophotometry following established methods^25^. PM_2.5_ filters from the MicroPEM were weighed before and after sampling at RTI International (USA), using a microbalance (Mettler Toledo UMX2) placed in an environmental chamber maintained at a temperature of 21 °C and 35% relative humidity. PM_2.5_ mass concentration was calculated by dividing PM mass collected during the week and measured by gravimetric analysis, by the air volume sampled during the measurement week (µg/m^3^). PM filters were cold-stored until OP analysis. The protocol for OP measurement was previously published^22,24^, based on the protocol established by Calas et al.^26,27^. Briefly, PM_2.5_ were extracted from filters into a simulated interstitial lung fluid consisting in a mixture of 1,2-dipalmitoylphosphatidylcholine (DPPC), to reach a final concentration of 10 µg/mL. Extracts were incubated at 37 °C for 75 min under vortex agitation prior to analysis using the dithiothreitol (DTT) and the ascorbic acid (AA) assays. A 96-well plate (CELLSTRAR, Greiner-Bio) was used to mix the extracts with DTT or AA solutions. For the AA assay, the absorbance at 265 nm (TECAN spectrophotometer Infinite M200 Pro) is measured over time to evaluate AA consumption by PM_2.5_ extract, for a total reaction time of 30 min. For the DTT assay, the absorbance at 412 nm measured the formation of the 2-nitro-5-thiobenzoic acid (TNB), which is the reaction product of the remaining DTT and dithionitrobenzoic acid (DTNB), for a total reaction time of 30 min. Samples were analyzed in triplicates, and the mean was calculated for each sample. For both assays, consumption rates were then normalized by the mass of PM of the extract (OP_m_, in nmol/min/µg), or by the corresponding air volume sampled (OP_v_, nmol/min/m^3^).

### Maternal immune function

Blood samples were collected by trained field workers, within a maximum of 48 h after the end of the exposure measurement week, following the procedure published by Manches et al.^20^. Briefly, blood was collected in BD Medical 368886 vacutainer tubes (lithium heparin) for immunological analyses (cell culture and plasma separation), and in BD Medical 368861 vacutainer tubes (EDTA) for cell counting. They were transported to the Etablissement Français du Sang (EFS) in coolers, placed on a rotating device for at least 5 min to ensure homogeneous cell content, and were then processed within 24 h after collection.

Innate and adaptative immunity of the women were measured at baseline and after a 24-h ex vivo activation of whole blood at 37 °C using dendritic cell activator Resiquimod (R848, InvivoGen, 5 μg/mL) and T cell activator phytohaemagglutinin (PHA, Oxoid, 10 μg/mL), as previously described by Manches et al.^20^.

Briefly, cytokines were measured in the culture supernatant (for activated cells) or in plasma (for baseline cytokines) by cytometric bead arrays (BD™ CBA Human cytokines Flex Set that is a bead-based immunoassay capable of simultaneously measuring several cytokines in biological fluids, BD Biosciences).

Among the 29 cytokines that were measured (12 at baseline, 9 after PHA-activation and 8 after R848 activation) only those with at least 70% of detected values were considered^20^. Hence, for the samples activated with PHA, the overall activity of T lymphocytes (T helpers Th1, Th2, Th9, Th17, and regulatory Treg) was assessed by quantifying the levels of IL-2, TNF-α, interferon (IFN) γ, IL-13, IL-17a, IL-9 and IL-10. For the samples activated with R848, the overall activity of dendritic cells was evaluated by quantifying TNF-α, IL-10, IL-6, IL-8, IFN-α, IFN-γ, IL-1β, and IL-12p70. For the non-activated sample, the basal state of the immune system was quantified by IL-8, monocyte chemoattractant protein-1 (MCP1) and regulated on activation, normal T cell expressed and secreted (RANTES). The concentrations below the limit of detection were imputed by a fill-in approach, that randomly selects values between 0 and the LOD based on the underlying distribution^28,29^. Due to their skewed distribution, cytokine concentrations were log10 transformed.

Since between-participant technical variability related to the experimentation can lead to measurement error, a two-step standardization method based on regression residuals^30^ was used to correct, when necessary, cytokine concentrations. The same standardized variables as previously described by Manches et al.^20^ were used. Briefly, the technical variables considered were: (1) for baseline cytokines: analytical batch, time between sample collection and reception, time between sample reception and analysis; (2) for activated cytokines the same variables were used, together with the duration of the activation, R848 or PHA age at the time of sample activation, and storage duration.

### Statistical analysis

Summary statistics (mean [sd] or median [Q1-Q3]) were calculated for air pollutant exposure assessments, cytokine levels, and covariates. A correlation matrix (Pearson’s *r*) was calculated between the cytokine levels and between the air pollutant concentrations. Univariate and adjusted linear regressions were conducted to estimate the associations between each air pollutant exposure and each cytokine level. Each exposure and log-transformed cytokine variable were divided by the interquartile range (IQR), to facilitate comparison of the beta estimates. Potential confounders were selected from the existing literature^31–33^, and using a directed acyclic graph (see Supplementary Fig. S1 online) and included: age of the women (continuous), BMI before pregnancy (continuous), active or passive smoking (active smoking in the 12 months prior to pregnancy, or active or passive smoking during pregnancy; binary: yes/no), educational level (binary: < master’s degree, ≥ master’s degree), leukocyte count (continuous), gestational age at sampling (continuous), and sampling season (4 categories with winter corresponding to January-March, spring to April-June, summer to July–September, and fall to October-December). To avoid reduction of the sample size due to missing data in cofactors (20 individuals had missing value for at least one covariate), multiple imputations (n = 20 datasets) were performed using Multivariate Imputation by Chained Equations (package mice, R).

In addition, sensitivity analyses were carried out to assess the robustness of the results to: (1) extreme values (after exclusion of 1% lowest and 1% highest exposure and cytokine concentrations), (2) influential values (after exclusion of values with a Cook’s distance exceeding 4/n, with n being the number of participants in the main analysis), (3) the set of confounders, with models excluding the leucocyte counts among cofactors and models including history of asthma and rhinitis which could lie in the causal path between air pollution and cytokine levels, and (4) considering bi-pollutant models, e.g. adjusting for NO_2_ in the PM_2.5_ and OP models, and adjusting for PM_2.5_ or OP in the NO_2_ models. Following statistical recommendations, interpretation of statistical tests was based on examining effects’ magnitude and their 95% confidence intervals and precise *p*-values (not whether *p*-values are above or below 0.05)^34,35^. All analyses were conducted using the statistical software R (version 4.2).

## Results

### Population characteristics

The population studied included 270 pregnant women with a median age of 32.1, a median BMI of 21.6 and a high educational level (58% had a diploma equivalent to or higher than a master’s degree) (Table 1). Among these women, 9.5% were active smokers before or during pregnancy. Regarding respiratory health, 16% reported asthma symptoms before or during pregnancy, and 40% reported rhinitis.

**Table 1:** Description of women in the study population.

Characteristics	N = 270^1^
Age (years)	32.1 (29.9–35.1)
Age (categories)	
< 30	69 (26%)
30–35	133 (49%)
≥ 35	68 (25%)
BMI before pregnancy	21.6 (19.8–24.1)
BMI before pregnancy (categories)	
< 18.5 kg/m^2^	15 (6%)
18.5–25 kg/m^2^	197 (74%)
25–30 kg/m^2^	43 (16%)
≥ 30 kg/m^2^	12 (4%)
Missing	3
Education level	
High school	13 (5%)
Bachelor’s degree	29 (11%)
Master’s degree	70 (26%)
≥ Postgraduate	157 (58%)
Missing	1
Active smoking before or during pregnancy	
No	228 (90%)
Yes	24 (10%)
Missing	18
Passive smoking during pregnancy	
No	204 (80%)
Yes	50 (20%)
Missing	16
Symptoms of asthma before or during pregnancy	
No	228 (84%)
Yes	42 (16%)
Symptoms of rhinitis before or during pregnancy	
No	162 (60%)
Yes	108 (40%)
Season of sampling	
Autumn	55 (20%)
Spring	83 (31%)
Summer	69 (26%)
Winter	63 (23%)
Gestational age at sampling (weeks)	19.0 (18.0–21.0)

^1^Median (Q1-Q3); n (%).

### ***Exposure to NO***_***2***_***, PM***_***2.5***_*** and OP***

**Fig. 2:**
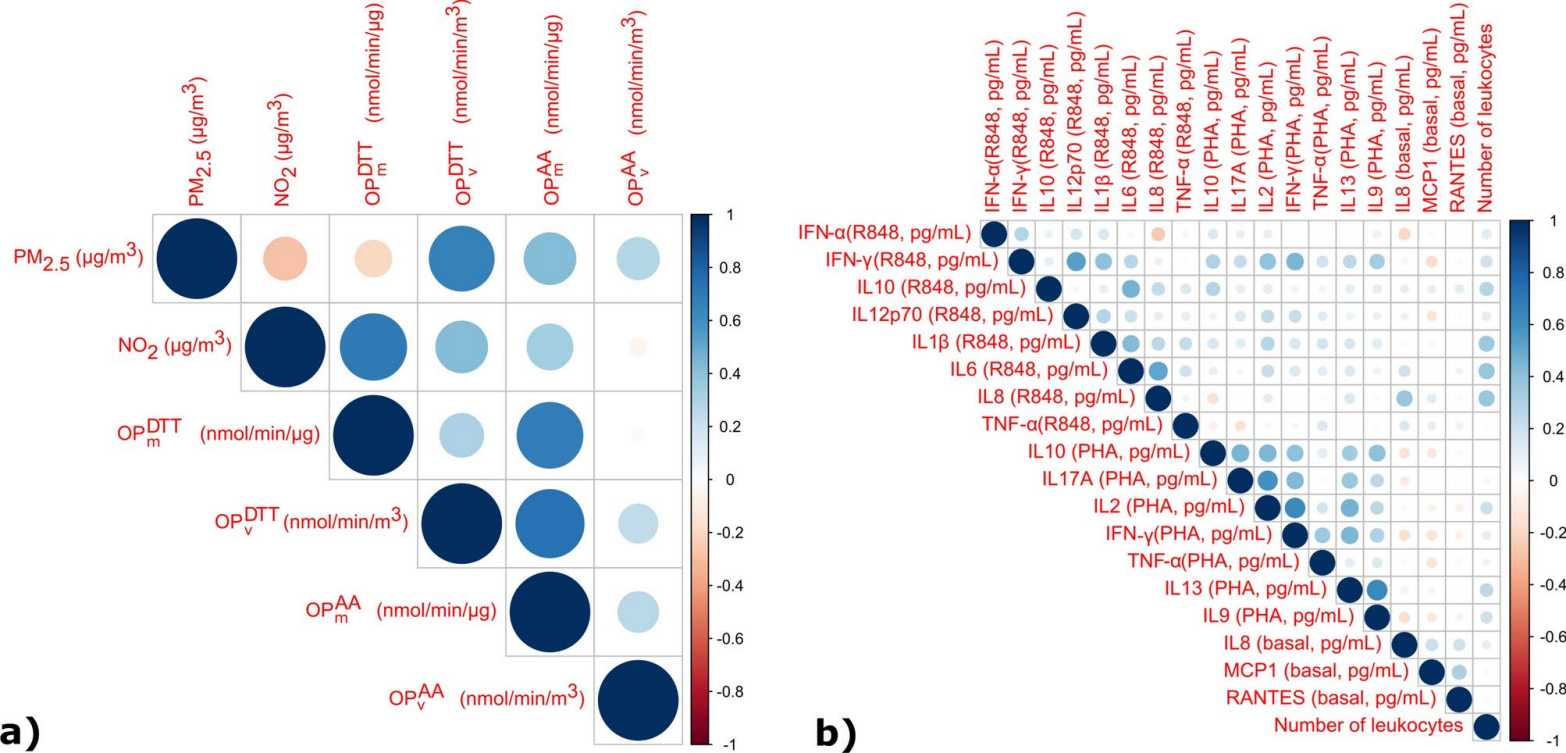
Pairwise Pearson’s correlations between air pollutants (**a**). Pairwise Pearson’s correlations between cytokines (imputed, corrected and log-10 transformed) and number of leukocytes (**b**). The Pearson correlation coefficients are indicated using a scale of size and color only when the *p*-values were less than 0.05. Abbreviations: PM_2.5_ particulate matter with diameter ≤ 2.5 μm; NO_2_ nitrogen dioxide; OP: oxidative potential; DTT: dithiothreitol; AA: ascorbic acid; IL interleukin; IFN interferon; TNF tumor necrosis factor; RANTES regulated on activation, normal T cell expressed and secreted; MCP monocyte chemoattractant protein; PHA phytohemagglutinin; R848 resiquimod

Median (Q1-Q3) exposure to NO_2_, PM_2.5_, ${\text{OP}}_{\text{m}}^{\text{DTT}}$, ${\text{OP}}_{\text{v}}^{\text{DTT}}$, ${\text{OP}}_{\text{m}}^{\text{AA}}$ and ${\text{OP}}_{\text{v}}^{\text{AA}}$ were 20.2 (16.2–25.8) µg/m^3^, 13.8 (9.9–18.5) µg/m^3^, 0.11 (0.09–0.14) nmol/min/µg, 1.48 (1.06–2.00) nmol/min/m^3^, 0.12 (0.08–0.16) nmol/min/µg and 1.65 (0.98–2.63) nmol/min/m^3^, respectively (Table 2). A seasonal trend was observed during the cold season with higher concentrations of all studied pollutants. ${\text{OP}}_{\text{v}}^{\text{AA}}$ presents a strong Pearson correlation coefficient (*r* > 0.5) with ${\text{OP}}_{\text{v}}^{\text{DTT}}$ and with ${\text{OP}}_{\text{m}}^{\text{AA}}$, whereas ${\text{OP}}_{\text{v}}^{\text{DTT}}$ is strongly correlated with PM_2.5_; and ${\text{OP}}_{\text{m}}^{\text{AA}}$ with ${\text{OP}}_{\text{m}}^{\text{DTT}}$ (Fig. 2). Moderate Pearson correlation coefficients (0.30 < *r* < 0.50) were observed between ${\text{OP}}_{\text{v}}^{\text{DTT}}$ and ${\text{OP}}_{\text{m}}^{\text{DTT}}$ or ${\text{OP}}_{\text{m}}^{\text{AA}}$, and between ${\text{OP}}_{\text{v}}^{\text{AA}}$ and PM_2.5_. Remaining correlations were considered weak (*r* < 0.3).

**Table 2:** Description of air pollutant characteristics.

Air pollutant	N	Median (Q1-Q3)	IQR
NO_2_ (µg/m^3^)	270	20.2 (16.2–25.8)	9.6
PM_2.5_ (µg/m^3^)	210	13.8 (9.9–18.5)	8.6
${\text{OP}}_{\text{m}}^{\text{DTT}}$(nmol/min/µg)	194	0.11 (0.09–0.14)	0.05
${\text{OP}}_{\text{v}}^{\text{DTT}}$(nmol/min/m^3^)	194	1.48 (1.06–2.00)	0.94
${\text{OP}}_{\text{m}}^{\text{AA}}$(nmol/min/µg)	194	0.12 (0.08–0.16)	0.08
${\text{OP}}_{\text{v}}^{\text{AA}}$(nmol/min/m^3^)	194	1.65 (0.98–2.63)	1.65

Abbreviations: IQR interquartile range; PM_2.5_ particulate matter with diameter ≤ 2.5 μm; NO_2_ nitrogen dioxide; OP: oxidative potential; DTT: dithiothreitol; AA: ascorbic acid.

### Cytokine levels

Regarding raw concentrations of cytokines, women exhibited median concentrations ranging from 17.8 (IL-8) to 14,026.3 (RANTES) pg/mL at the basal state, from 35.8 (IL-9) to 2664.7 (IL-2) pg/mL after PHA activation, and from 22.5 (IL-12p70) to 44,667.9 (IL-6) after R848 activation (Table 3). Regarding imputed, corrected and log-10 transformed cytokine concentrations, the intra-group (basal, PHA activated, R848 activated) correlations at the basal level displayed a low correlation (−0.1 < Pearson r < 0.2), correlations after PHA activation were moderate-to-strong (*r* > 0.4) and correlations after R848 activation were weak-to-moderate (0.2 < *r* < 0.4) (Fig. 2). The inter-group correlation was very weak (*r* < 0.1), except for IL-8 between basal state and R848-activated measures (*r* = 0.37), IFN-γ between PHA- and R848- activated measures (*r* = 0.44) and between PHA-activated IL-2 and R848-activated IL-6, IL-1β, and IL-12p70 (0.22 < *r* < 0.27). A negative correlation was also observed between IL-10 secretion after PHA activation and IL-8 secretion at basal state and after R848 activation (*r* = −0.15 and −0.16, respectively).

**Table 3:** Description of biomarkers characteristics.

Cytokine levels	Percentile of raw values						Percentile of imputed, corrected and log10 transformed values					
	N	10%	25%	50%	75%	90%	10%	25%	50%	75%	90%	IQR
Concentration of leucocytes (G/L)	265	6.8	7.6	8.9	10.2	11.6						
At basal state												
IL-8 (pg/mL)	266	5.5	8.9	17.8	94.3	283.1	0.50	0.84	1.07	1.36	1.86	0.52
MCP1 (pg/mL)	266	19.0	25.9	38.4	56.2	116.0	1.22	1.38	1.51	1.64	1.77	0.26
RANTES (pg/mL)	266	7374.1	9873.3	14,026.3	17,511.4	23,935.5	3.84	3.96	4.08	4.16	4.24	0.20
After PHA activation												
IFN-γ (pg/mL)	264	300.0	429.7	758.4	1383.8	3121.9	2.60	2.75	2.94	3.14	3.30	0.39
IL-2 (pg/mL)	264	1067.1	1701.6	2664.7	4245.8	7877.3	3.13	3.30	3.47	3.65	3.78	0.35
IL-9 (pg/mL)	264	13.8	20.7	35.8	59.7	86.8	1.29	1.45	1.64	1.85	2.05	0.40
IL-10 (pg/mL)	264	428.4	609.1	904.3	1369.6	2428.1	2.76	2.91	3.04	3.18	3.30	0.27
IL-13 (pg/mL)	264	57.1	83.9	138.1	205.8	339.4	1.75	1.93	2.11	2.26	2.40	0.33
IL-17A (pg/mL)	259	94.0	156.6	282.0	518.6	1151.4	2.15	2.32	2.55	2.78	2.97	0.43
TNF-α (pg/mL)	264	12.8	247.9	760.2	1468.2	2489.4	1.38	2.22	2.70	2.97	3.22	0.75
After R848 activation												
IFN-α (pg/mL)	270	41.6	116.7	259.2	573.1	976.9	2.20	2.39	2.68	2.92	3.12	0.53
IFN-γ (pg/mL)	270	82.3	173.0	366.8	738.5	1211.7	1.93	2.24	2.60	2.89	3.16	0.65
IL-1β (pg/mL)	270	4503.4	6481.8	9157.4	13,545.0	19,700.1	3.60	3.73	3.88	4.02	4.15	0.29
IL-6 (pg/mL)	270	21,720.3	32,069.2	44,667.9	60,509.2	87,701.3	4.26	4.40	4.54	4.66	4.79	0.26
IL-8 (pg/mL)	270	12,534.4	20,895.5	39,810.9	66,939.8	112,322.1	3.93	4.10	4.35	4.54	4.76	0.44
IL-10 (pg/mL)	270	348.4	530.7	782.9	1090.5	1348.7	2.51	2.66	2.79	2.91	3.05	0.25
IL-12p70 (pg/mL)	270	7.1	11.7	22.5	37.5	61.0	0.87	1.11	1.35	1.59	1.79	0.48
TNF-α (pg/mL)	270	116.3	2643.8	6382.1	11,480.7	15,127.2	1.84	2.82	3.21	3.44	3.66	0,62

Abbreviations: IL, interleukin; IFN interferon; TNF tumor necrosis factor; RANTES regulated on activation, normal T cell expressed and secreted; MCP monocyte chemoattractant protein; PHA phytohemagglutinin; R848 resiquimod.

### Associations between personal exposures to air pollutants and immune function parameters

**Fig. 3:**
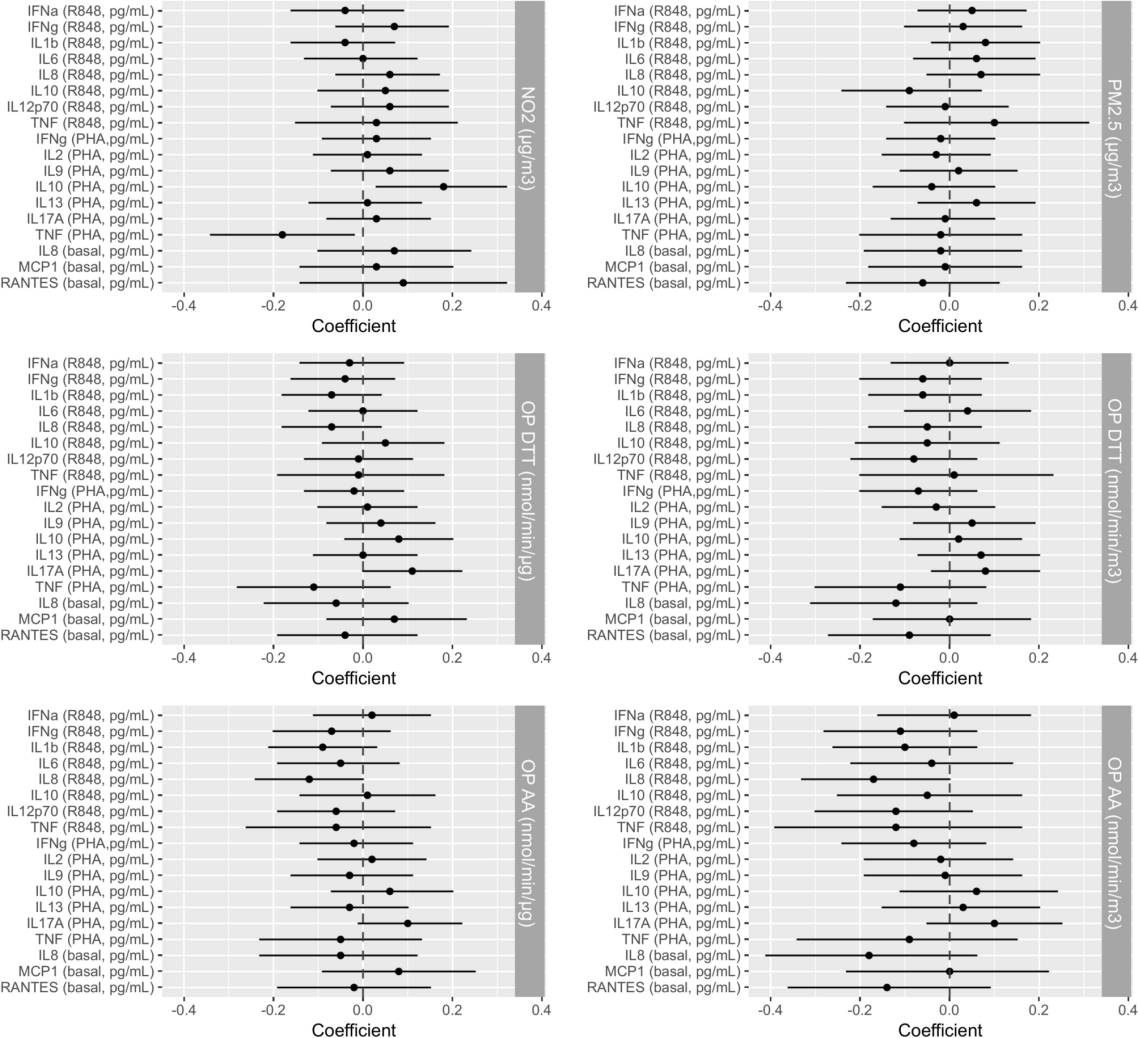
Adjusted associations between each immunological parameter and each personal exposure to NO_2_, PM_2.5_, OP^DTT^ and OP^AA^ during pregnancy. Pollutants and cytokines variables were standardized by their IQR. Beta values and their 95% CI were estimated by multiple linear regression models. Models were adjusted on women age, BMI, active or passive smoking, educational level, white blood cell count, gestational age at sampling, and sampling season. Abbreviations: PM_2.5_ particulate matter with diameter ≤ 2.5 μm; NO_2_ nitrogen dioxide; OP oxidative potential; DTT, dithiothreitol; AA, ascorbic acid; CI, confidence interval; IQR, interquartile; BMI, body mass index; Il, interleukin; IFN interferon; TNF tumor necrosis factor; RANTES regulated on activation, normal T cell expressed and secreted; MCP monocyte chemoattractant protein; PHA phytohemagglutinin; R848 resiquimod.

Univariate analyses are presented in Supplementary Tables online. In multivariate analyses (Fig. 3), a 10 µg/m3 (IQR) increase in NO_2_ exposure was associated with higher PHA-activated IL-10 and lower PHA-activated TNF-α (β [95% CI] = 0.18 [0.03, 0.32], *p* = 0.02; β [95% CI] = −0.18 [−0.32, −0.02], *p* = 0.03, respectively). No association was observed with PM_2.5_ concentration, but increased exposure to PM ${\text{OP}}_{\text{v}}^{\text{AA}}$ (IQR = 1.65 nmol/min/m^3^) was associated with lower IL-8 measured upon R848 activation (β [95% CI] = −0.17 [−0.33, 0.00], *p* = 0.05), and a similar trend was observed for basal IL-8 levels (β [95% CI] = −0.18 [−0.41, 0.06], *p* = 0.14). An IQR-increase in exposure to ${\text{OP}}_{\text{m}}^{AA}$ (IQR = 0.08 nmol/min/µg) was associated with lower R848-activated IL-8 (β [95% CI] = −0.12 [−0.24, 0.00], *p* = 0.05). Finally, ${\text{OP}}_{\text{m}}^{\text{DTT}}$ was associated with higher PHA-activated IL-17A (β [95% CI] = 0.11 [0.00, 0.22], *p* = 0.04), and a similar trend of association was observed for ${\text{OP}}_{\text{v}}^{\text{DTT}}$ and ${\text{OP}}_{\text{m}}^{AA}$ (β [95% CI] = 0.08 [−0.04, 0.20], *p* = 0.18; β [95% CI] = 0.10 [−0.01, 0.22], *p* = 0.07, respectively) (see Supplementary Tables online).

Overall, results were robust, as displayed by the sensitivity analyses. Only the association between NO_2_ and PHA activated IL-10 disappeared with the exclusion of extreme and influential values and in the models adjusted on PM or OP. Other findings were either similar, or stronger, as for the NO_2_-TNF association (see Supplementary Fig. S2 online). The other models excluding the leucocyte counts among cofactors, and including history of asthma and rhinitis, provided the same results as the main models.

## Discussion

### Comparison with others studies

To our knowledge, this is the first study investigating the association between personal exposures to PM_2.5_, OP of PM_2.5_, and NO_2_, with basal and activated immune function parameters in pregnant women. For PM_2.5_ and NO_2_, personal exposure levels are in the range of typical ambient exposure levels, as reported in the ELAPSE project, pooling eight European cohorts^36^. Our results show that increase in NO_2_ exposure was associated with higher PHA-activated TNF–α. Similarly, an increase in ${\text{OP}}_{\text{m}}^{\text{AA}}$, as well as in ${\text{OP}}_{\text{v}}^{\text{AA}}$, led to increase in R848-activated IL-8. Finally, a positive association was suggested between PHA-activated IL-17A with the three OP measurements. Interestingly, the study did not find any significant associations with PM_2.5_, but it did reveal significant associations with the capacity of PM to induce oxidative stress, as measured by OP.

The mechanisms involved in the health impacts of air pollutants include impairment of the innate and adaptative immunity, and the activation of oxidative stress and reactive oxygen species^37^. Adaptive immunity and oxidative stress closely interact with each other, these two mechanisms being triggered independently but having effects on each other. By investigating the effects of NO_2_, PM_2.5_ and OP of PM_2.5_ exposure on immunological parameters, our study was able to provide a complete picture of the cross-interaction of oxidative stress and adaptative immunity pathways.

The results observed in the current study, showing decreased TNF-α levels upon NO_2_ exposure, are in line with previous research in non-pregnant adults^31^. However, positive associations between PM_2.5_ and TNF-α reported in previous studies^32,33,38,39^ were not observed in our study. Potential differences in study populations, measurement methodologies and analysis models could play a role in these disparities. More specifically, Chen et al.^38^ investigated these associations in students and the three other studies (Gong et al.^39^, Friedman et al.^32^ and Zhang et al.^33^) were based on a population of pregnant women but used ambient exposure (not personal exposure assessment), and Gong et al.^39^ examined cytokine levels in the placenta, which could lead to different results compared to blood.

Very few studies have addressed the effects of OP exposure on immune response in human blood, and when they did, IL-6 levels were mostly investigated^14,16^. In the RAPTES project^16^, blood and nasal lavage from 31 healthy student volunteers were retrieved after 5 h of exposure in different ambient settings, with contrasted pollution levels. No effect was observed with any of the analyzed OP tests (AA and Glutathione assays). The observed negative OP-IL-8 association is consistent with some previous findings among non-pregnant individuals that considered PM_2.5_ exposure from one week^31^ to three months^40^ and from both indoor^40^ and ambient environments^31,41,42^. Contradictory, positive IL-8-PM_2.5_ mass and IL-8-NO_2_ relationships were reported by some experimental studies^43–45^, which may differ from real environmental contexts in human studies.

The results of our study indicate a positive association between OP exposure and IL-17A. To the best of our knowledge, no prior research has specifically examined this relationship. Previous studies used NO_2_ or PM_2.5_ exposure to examine effects on IL-17A in non-pregnant participants, and similar positive associations were reported with PM_2.5_^46^ and NO_2_ exposure^31,41,46^. The study conducted by Hu et al.^31^ addressed these associations by varying the durations of exposure to PM_2.5_ and NO_2_, which led to contrasting observations. Specifically, a short-term exposure to NO_2_ (between 12 and 24 h) was significantly correlated with high levels of IL-17A, whereas prolonged exposure (two weeks) was statistically associated with reduced levels of IL-17A. Our study extends these findings that relied on ambient PM_2.5_ and NO_2_ exposure, by using personal exposure assessment, by measuring PM_2.5_ OP exposure, and by activating the cells.

Our study presents the specificity of analyzing cytokines in pregnant women, who present several variations of immunity compared to non-pregnant women. In a recent study^47^, at basal state, most inflammatory cytokines among which TNF-α and IL-8 decreased in the second trimester. We observed here at the same period of pregnancy a global trend towards a decrease of these cytokines at basal state and after activation of immune cells, suggesting that there might be a cumulative negative effect of pregnancy and exposure to air pollutants on inflammatory cytokine secretions that could impair maternal health and capacity to respond to pathogens. Moreover, we observed a positive association between NO_2_ exposure and IL-10 secretion upon activation with PHA, which could participate in the reduction of inflammatory cytokines that was detected here. Indeed, IL-10 has broad regulatory effects on several immune cells, and is involved in normal pregnancy processes of tolerance^48^. In our experimental settings where whole blood cells are activated by PHA, IL-10 may be produced by regulatory T cells that are also involved in tolerance mechanisms towards the fetus during pregnancy^49^.

Overall, our results on the impact of air pollution exposure on the inflammatory function of the immune system may provide insights into the mechanisms underlying the adverse health effects of air pollution. In particular, IL-17A secretion is strongly linked to severe forms of asthma^50,51^, hypertension during pregnancy^52^ and changes in birth weight^53^. T lymphocytes, producers of IL-17A, are also pathogenic cellular components of autoimmune diseases, such as multiple sclerosis or psoriasis^52^. Circulating IL-17A decreases during pregnancy^47^, and our results suggest that air pollution could interact synergistically with this regulation of Th17 cells, with potential consequences on maternal health. In addition, TNF-α has also been identified as playing a role in the inflammatory response in allergies, which are related to air pollution^54^.

### Strengths and limitations

One of the primary strengths of this study lies in its meticulous exposure measurements. Personal exposure measurements provided accurate assessment, tailored to the individual, in contrast to conventional assessments based on ambient exposure data obtained from monitoring stations or exposure models. The potential effect of a peak exposure over a short period of the week is diluted in our study, as we used particle samples integrated over 7 days. Consequently, the associations reported in our study may be underestimated. However, pregnant women are inclined to spend a greater portion of their time indoors, where air pollutants sources and chemical components differ from the ambient environment; personal exposure measurements are therefore all the more important. Another asset is that the study focuses not only on cytokines circulating at basal level, but also on cytokine levels measured after activation of innate and T cells. Consequently, the results obtained closely approximate real immune cell functionality, reflecting the actual immune system response to aggression in the context of potential damages induced by air pollution exposure. Analyses on cytokine levels after activation could potentially reduce confusion bias compared to analyses considering cytokine basal levels since participants’ cells were activated using the same procedure. The main asset of this work is probably the use of OP measurements, that aims to account for the detrimental impacts of PM_2.5_ through the oxidative stress pathway, and this led to clearer associations compared to the mass concentration metric. Furthermore, since the main sources contributing to PM and to the OP of PM were already reported in the Grenoble area^55^, it is particularly relevant to examine both factors in the current Grenoble-based study.

Although the study is based on an a priori hypothesis, the number of associations tested is relatively high and we have not applied a formal correction for multiple comparisons. It should therefore be recognized that some of the associations identified could result from chance finding and should therefore be interpreted with caution. The relatively small sample size of this study limits the statistical power and the possibility to robustly investigate differences in the effects of air pollution in the immune function by the asthma/rhinitis status. A larger population would potentially lead to more robust conclusions. However, this is counterbalanced by the accuracy of the measurements through the use of personal samplers, that significantly decreases measurement error. A further constraint of this study relates to the recruited population in SEPAGES, which does not reflect the overall diversity of the general population. This study is geographically restricted to the Grenoble region with a specific semi-continental climate and orography that lead to important thermal-inversions in the winter season, thereby increasing ground concentrations of pollutants. This drawback is compensated by the fact that such concentrations are nevertheless quite common in Europe, and concern a large share of the population. In addition, the participants included had higher levels of education and were more often non-smoker, as compared to pregnant women in France and some potential confounders were not taken into account, e.g. gas or electric cooking, heating system. Nevertheless, analyses in this homogeneous population are less prone to confounding biases related to social environment. Lastly, the conducted study specifically focuses on the independent effects of each pollutant on each included cytokine. The bi-pollutant models provided additional support to the observed association. Potential synergistic effects between pollutants would require a larger sample size to be robustly tested. This might be considered as simplistic, because interaction and cumulative effect of various air pollutants are expected, and cytokines do not operate in isolation within the immune system but are involved in a complex network of regulations and interactions of the body. However, studying the isolated effects provides a solid grasp of the underlying mechanisms, even though all intricacies of the system are not captured.

## Conclusion

In conclusion, our study provides significant insights into the impact of exposure to air pollutants, including oxidative potential of PM, on immune function among pregnant women. This crucial data can be instrumental in creating strategies to reduce the oxidative potential of PM_2.5_ and thus to mitigate the adverse health effects of air pollution.

## Supplementary Information

Supplementary Information 1.

Supplementary Information 2.

## Data Availability

All data generated or analyzed during this study are available upon reasonable request from the corresponding author.
