# Supplementary material for: Personal exposure to air pollutants and immune system biomarkers in pregnant women
Source: Sci Rep. 2025 May 21;15:17672. doi: 10.1038/s41598-025-98712-7 (PMC12095663; doi:10.1038/s41598-025-98712-7)
Supplement: Supplementary file 1 — Supplementary Information 1. [file 41598_2025_98712_MOESM1_ESM.docx]

Personal exposure to air pollutants and immune system biomarkers in pregnant women – Supplementary information

Anouk Marsal^1,2,8,#^, Laurene Frau^2,#^, Laurence Chaperot^2,3^, Ines Amine^2^, Sarah Lyon-Caen^2^, Anne Boudier^2,4^, Jean-Luc Jaffrezo^1^, Rhabira Elazzouzi^1^, Claire Philippat^2^, Karine Supernant^2^, Johanna Lepeule^2^, Joane Quentin^2,5^, Ryan Chartier^6^, Sam Bayat^5,7^, Remy Slama^2^, Gaelle Uzu^1^, Valérie Siroux^2,*^

^1^ Univ. Grenoble Alpes, CNRS, INRAE, IRD, Grenoble INP, IGE, 38000 Grenoble, FRANCE.

^2^ Univ. Grenoble-Alpes, INSERM U1209, CNRS UMR 5309, Institut pour l’Avancée des Biosciences (IAB), Team of environmental epidemiology applied to development and respiratory health 38000 Grenoble, France.

^3^ EFS, Recherche et Développement, 38000 Grenoble, France.

^4^ Pediatric Department, CHU Grenoble Alpes, Grenoble, France.

^5^ Department of Pulmonology and Physiology, CHU Grenoble Alpes, Grenoble, France.

^6^ RTI International, Research Triangle Park, Durham, North Carolina, USA.

^7^ Univ. Grenoble Alpes, Inserm UA07 STROBE Laboratory, Grenoble, France.

^8^ Agence de l’environnement et de la Maîtrise de l’Energie. 20, avenue du Grésillé- BP 90406 49004 Angers Cedex 01, France.

9Univ. Grenoble Alpes, Inserm U1209, CNRS UMR 5309, Institut pour l’Avancée des Biosciences, Team of Epigenetics, Immunity, Metabolism, Cell Signaling & Cancer, 38000 Grenoble, France.

#: co-first authors who participated equally to this work

*: corresponding author: Valérie Siroux (valerie.siroux@univ-grenoble-alpes.fr)

### List of Figures

[Figure S1. Directed acyclic graph (DAG) mapping causal relationships 2](#_Toc187337690)

[Figure S2. Sensitivity analyses of the associations between NO_2_ and cytokine levels. 6](#_Toc187337691)

[Figure S3. Sensitivity analyses of the associations between PM_2.5_ and cytokine levels. 7](#_Toc187337692)

[Figure S4. Sensitivity analyses of the associations between OP_v_^AA^ and cytokine levels. 8](#_Toc187337693)

[Figure S5. Sensitivity analyses of the associations between OP_m_^AA^ and cytokine levels. 9](#_Toc187337694)

[Figure S6. Sensitivity analyses of the associations between OP_v_^DTT^ and cytokine levels. 10](#_Toc187337695)

[Figure S7. Sensitivity analyses of the associations between OP_m_^DTT^ and cytokine levels. 11](#_Toc187337696)

### List of Tables

[Table S1. Associations between PM_2.5_, NO_2_ and each cytokine levels in the univariate and adjusted models. 3](#_Toc187337697)

[Table S2. Associations between OP_m_^DTT^, OP_v_^DTT^ and each cytokine levels in the univariate and adjusted models. 4](#_Toc187337698)

[Table S3. Associations between OP_m_^AA^, OP_v_^AA^ and each cytokine levels in the univariate and adjusted models. 5](#_Toc187337699)


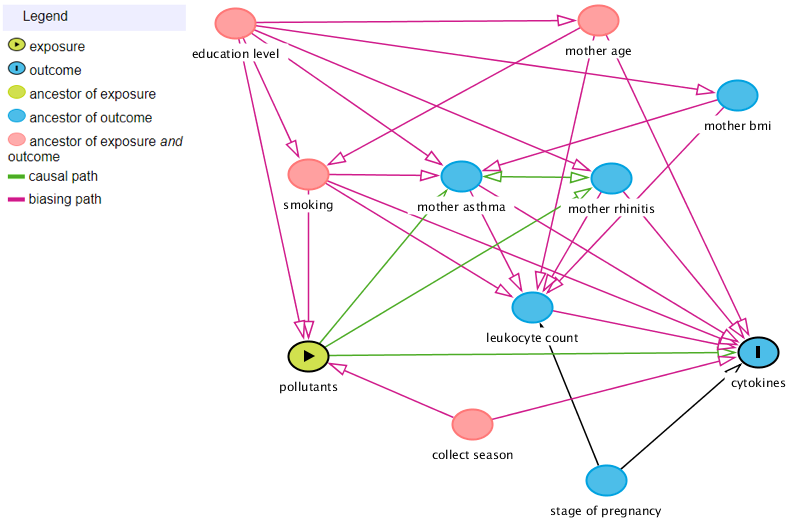


Figure S1. Directed acyclic graph (DAG) mapping causal relationships

Table S1. Associations between PM_2.5_, NO_2_ and each cytokine levels in the univariate and adjusted models.

|  | **NO_2_ (μg/m^3^)** | | | | | **PM_2.5_ (μg/m^3^)** | | | | |
| --- | --- | --- | --- | --- | --- | --- | --- | --- | --- | --- |
|  |  | **Univariate model** | | **Adjusted model** | |  | **Univariate model** | | **Adjusted model** | |
|  | **N** | **β** | **95% CI** | **β** | **95% CI** | **N** | **β** | **95% CI** | **β** | **95% CI** |
| **IFN-α R848 (pg/mL)** | 270 | 0.00 | [-0.13, 0.12] | -0.04 | [-0.16, 0.09] | 210 | 0.06 | [-0.06, 0.19] | 0.05 | [-0.07, 0.17] |
| **IFN-γ (R848, pg/mL)** | 270 | 0.08 | [-0.04, 0.20] | 0.07 | [-0.06, 0.19] | 210 | 0.01 | [-0.12, 0.14] | 0.03 | [-0.10, 0.16] |
| **IL-1β (R848, pg/mL)** | 270 | -0.03 | [-0.15, 0.09] | -0.04 | [-0.16, 0.07] | 210 | 0.07 | [-0.05, 0.20] | 0.08 | [-0.04, 0.20] |
| **IL-6 (R848, pg/mL)** | 270 | -0.01 | [-0.15, 0.12] | 0.00 | [-0.13, 0.12] | 210 | 0.03 | [-0.11, 0.17] | 0.06 | [-0.08, 0.19] |
| **IL-8 (R848, pg/mL)** | 270 | 0.04 | [-0.08, 0.17] | 0.06 | [-0.06, 0.17] | 210 | 0.03 | [-0.10, 0.16] | 0.07 | [-0.05, 0.20] |
| **IL-10 (R848, pg/mL)** | 270 | 0.05 | [-0.10, 0.21] | 0.05 | [-0.10, 0.19] | 210 | -0.09 | [-0.25, 0.07] | -0.09 | [-0.24, 0.07] |
| **IL-12p70 (R848, pg/mL)** | 270 | 0.07 | [-0.05, 0.19] | 0.06 | [-0.07, 0.19] | 210 | 0.01 | [-0.12, 0.13] | -0.01 | [-0.14, 0.13] |
| **TNF-α (R848, pg/mL)** | 270 | 0.01 | [-0.16, 0.19] | 0.03 | [-0.15, 0.21] | 210 | 0.09 | [-0.10, 0.28] | 0.10 | [-0.10, 0.31] |
| **IFN-γ (PHA, pg/mL)** | 264 | 0.04 | [-0.08, 0.16] | 0.03 | [-0.09, 0.15] | 204 | -0.02 | [-0.14, 0.09] | -0.02 | [-0.14, 0.10] |
| **IL-2 (PHA, pg/mL)** | 264 | 0.03 | [-0.09, 0.16] | 0.01 | [-0.11, 0.13] | 204 | 0.00 | [-0.12, 0.13] | -0.03 | [-0.15, 0.09] |
| **IL-9 (PHA, pg/mL)** | 264 | 0.09 | [-0.04, 0.21] | 0.06 | [-0.07, 0.19] | 204 | 0.04 | [-0.09, 0.17] | 0.02 | [-0.11, 0.15] |
| **IL-10 (PHA, pg/mL)** | 264 | 0.19 | [0.04, 0.33] | 0.18 | [0.03, 0.32] | 204 | 0.00 | [-0.13, 0.13] | -0.04 | [-0.17, 0.10] |
| **IL-13 (PHA, pg/mL)** | 264 | 0.04 | [-0.09, 0.17] | 0.01 | [-0.12, 0.13] | 204 | 0.08 | [-0.05, 0.21] | 0.06 | [-0.07, 0.19] |
| **IL-17A (PHA, pg/mL)** | 259 | 0.06 | [-0.06, 0.18] | 0.03 | [-0.08, 0.15] | 199 | 0.04 | [-0.08, 0.16] | -0.01 | [-0.13, 0.10] |
| **TNF-α (PHA, pg/mL)** | 264 | -0.18 | [-0.34, -0.03] | -0.18 | [-0.34, -0.02] | 204 | -0.05 | [-0.22, 0.13] | -0.02 | [-0.20, 0.16] |
| **IL-8 (basal, pg/mL)** | 266 | 0.03 | [-0.14, 0.20] | 0.07 | [-0.10, 0.24] | 209 | -0.05 | [-0.23, 0.12] | -0.02 | [-0.19, 0.16] |
| **MCP-1 (basal, pg/mL)** | 266 | 0.03 | [-0.13, 0.19] | 0.03 | [-0.14, 0.20] | 209 | -0.03 | [-0.19, 0.13] | -0.01 | [-0.18, 0.16] |
| **RANTES (basal, pg/mL)** | 266 | 0.09 | [-0.14, 0.32] | 0.09 | [-0.14, 0.32] | 209 | -0.07 | [-0.23, 0.10] | -0.06 | [-0.23, 0.11] |

*Pollutants and cytokines variables were standardized by IQR. Adjusted models were adjusted on mother age, BMI, active or passive smoking, educational level, white blood cell count, gestational age at sampling, and sampling season.*

*Abbreviations: NO_2_ nitrogen dioxide, PM_2.5_ particulate matter with diameter ≤ 2.5 μm, CI, confidence interval, IL, interleukin, IFN interferon, TNF tumor necrosis factor, RANTES regulated on activation, normal T cell expressed and secreted, MCP monocyte chemoattractant protein.*

Table S2. Associations between OP_m_^DTT^, OP_v_^DTT^ and each cytokine levels in the univariate and adjusted models.

|  | **OP_m_^DTT^ (nmol/min/μg)** | | | | | **OP_v_^DTT^ (nmol/min/m^3^)** | | | | |
| --- | --- | --- | --- | --- | --- | --- | --- | --- | --- | --- |
|  |  | **Univariate model** | | **Adjusted model** | |  | **Univariate model** | | **Adjusted model** | |
|  | **N** | **β** | **95% CI** | **β** | **95% CI** | **N** | **β** | **95% CI** | **β** | **95% CI** |
| **IFN-α R848 (pg/mL)** | 194 | -0.01 | [-0.12, 0.11] | -0.03 | [-0.14, 0.09] | 194 | 0.05 | [-0.08, 0.18] | 0.00 | [-0.13, 0.13] |
| **IFN-γ (R848, pg/mL)** | 194 | -0.04 | [-0.15, 0.08] | -0.04 | [-0.16, 0.07] | 194 | -0.06 | [-0.19, 0.07] | -0.06 | [-0.20, 0.07] |
| **IL-1β (R848, pg/mL)** | 194 | -0.05 | [-0.17, 0.07] | -0.07 | [-0.18, 0.04] | 194 | -0.02 | [-0.15, 0.11] | -0.06 | [-0.18, 0.07] |
| **IL-6 (R848, pg/mL)** | 194 | 0.00 | [-0.13, 0.13] | 0.00 | [-0.12, 0.12] | 194 | 0.02 | [-0.13, 0.16] | 0.04 | [-0.10, 0.18] |
| **IL-8 (R848, pg/mL)** | 194 | -0.05 | [-0.17, 0.08] | -0.07 | [-0.18, 0.04] | 194 | -0.06 | [-0.20, 0.07] | -0.05 | [-0.18, 0.07] |
| **IL-10 (R848, pg/mL)** | 194 | 0.06 | [-0.08, 0.21] | 0.05 | [-0.09, 0.18] | 194 | -0.02 | [-0.19, 0.14] | -0.05 | [-0.21, 0.11] |
| **IL-12p70 (R848, pg/mL)** | 194 | -0.01 | [-0.13, 0.11] | -0.01 | [-0.13, 0.11] | 194 | -0.06 | [-0.19, 0.07] | -0.08 | [-0.22, 0.06] |
| **TNF-α (R848, pg/mL)** | 194 | 0.00 | [-0.19, 0.18] | -0.01 | [-0.19, 0.18] | 194 | 0.00 | [-0.20, 0.20] | 0.01 | [-0.20, 0.23] |
| **IFN-γ (PHA, pg/mL)** | 188 | -0.01 | [-0.12, 0.10] | -0.02 | [-0.13, 0.09] | 188 | -0.05 | [-0.17, 0.07] | -0.07 | [-0.20, 0.06] |
| **IL-2 (PHA, pg/mL)** | 188 | 0.02 | [-0.10, 0.13] | 0.01 | [-0.10, 0.12] | 188 | 0.04 | [-0.09, 0.16] | -0.03 | [-0.15, 0.10] |
| **IL-9 (PHA, pg/mL)** | 188 | 0.06 | [-0.06, 0.18] | 0.04 | [-0.08, 0.16] | 188 | 0.10 | [-0.04, 0.23] | 0.05 | [-0.08, 0.19] |
| **IL-10 (PHA, pg/mL)** | 188 | 0.09 | [-0.03, 0.21] | 0.08 | [-0.04, 0.20] | 188 | 0.08 | [-0.05, 0.21] | 0.02 | [-0.11, 0.16] |
| **IL-13 (PHA, pg/mL)** | 188 | 0.02 | [-0.11, 0.14] | 0.00 | [-0.11, 0.12] | 188 | 0.12 | [-0.02, 0.25] | 0.07 | [-0.07, 0.20] |
| **IL-17A (PHA, pg/mL)** | 183 | 0.10 | [-0.01, 0.21] | 0.11 | [0.00, 0.22] | 183 | 0.14 | [0.02, 0.26] | 0.08 | [-0.04, 0.20] |
| **TNF-α (PHA, pg/mL)** | 188 | -0.11 | [-0.27, 0.05] | -0.11 | [-0.28, 0.06] | 188 | -0.13 | [-0.31, 0.04] | -0.11 | [-0.30, 0.08] |
| **IL-8 (basal, pg/mL)** | 193 | -0.07 | [-0.23, 0.09] | -0.06 | [-0.22, 0.10] | 193 | -0.18 | [-0.36, -0.01] | -0.12 | [-0.31, 0.06] |
| **MCP-1 (basal, pg/mL)** | 193 | 0.08 | [-0.07, 0.23] | 0.07 | [-0.08, 0.23] | 193 | 0.00 | [-0.17, 0.17] | 0.00 | [-0.17, 0.18] |
| **RANTES (basal, pg/mL)** | 193 | -0.01 | [-0.17, 0.14] | -0.04 | [-0.19, 0.12] | 193 | -0.07 | [-0.24, 0.10] | -0.09 | [-0.27, 0.09] |

*Pollutants and cytokines variables were standardized by IQR. Adjusted models were adjusted on mother age, BMI, active or passive smoking, educational level, white blood cell count, gestational age at sampling, and sampling season.*

*Abbreviations: OP: oxidative potential, DTT: dithiothreitol, CI, confidence interval, IL, interleukin, IFN interferon, TNF tumor necrosis factor, RANTES regulated on activation, normal T cell expressed and secreted, MCP monocyte chemoattractant protein.*

Table S3. Associations between OP_m_^AA^, OP_v_^AA^ and each cytokine levels in the univariate and adjusted models.

|  | **OP_m_^AA^ (nmol/min/μg)** | | | | | **OP_v_^AA^ (nmol/min/m^3^)** | | | | |
| --- | --- | --- | --- | --- | --- | --- | --- | --- | --- | --- |
|  |  | **Univariate model** | | **Adjusted model** | |  | **Univariate model** | | **Adjusted model** | |
|  | **N** | **β** | **95% CI** | **β** | **95% CI** | **N** | **β** | **95% CI** | **β** | **95% CI** |
| **IFN-α R848 (pg/mL)** | 194 | 0.08 | [-0.04, 0.20] | 0.02 | [-0.11, 0.15] | 194 | 0.10 | [-0.05, 0.25] | 0.01 | [-0.16, 0.18] |
| **IFN-γ (R848, pg/mL)** | 194 | -0.04 | [-0.16, 0.08] | -0.07 | [-0.20, 0.06] | 194 | -0.07 | [-0.22, 0.08] | -0.11 | [-0.28, 0.06] |
| **IL-1β (R848, pg/mL)** | 194 | -0.06 | [-0.18, 0.06] | -0.09 | [-0.21, 0.03] | 194 | -0.06 | [-0.21, 0.09] | -0.10 | [-0.26, 0.06] |
| **IL-6 (R848, pg/mL)** | 194 | -0.09 | [-0.22, 0.04] | -0.05 | [-0.19, 0.08] | 194 | -0.10 | [-0.27, 0.06] | -0.04 | [-0.22, 0.14] |
| **IL-8 (R848, pg/mL)** | 194 | -0.14 | [-0.27, -0.02] | -0.12 | [-0.24, 0.00] | 194 | -0.21 | [-0.36, -0.05] | -0.17 | [-0.33, 0.00] |
| **IL-10 (R848, pg/mL)** | 194 | 0.01 | [-0.14, 0.17] | 0.01 | [-0.14, 0.16] | 194 | -0.04 | [-0.23, 0.15] | -0.05 | [-0.25, 0.16] |
| **IL-12p70 (R848, pg/mL)** | 194 | -0.04 | [-0.15, 0.08] | -0.06 | [-0.19, 0.07] | 194 | -0.07 | [-0.22, 0.08] | -0.12 | [-0.30, 0.05] |
| **TNF-α (R848, pg/mL)** | 194 | -0.08 | [-0.27, 0.11] | -0.06 | [-0.26, 0.15] | 194 | -0.13 | [-0.36, 0.10] | -0.12 | [-0.39, 0.16] |
| **IFN-γ (PHA, pg/mL)** | 188 | 0.01 | [-0.10, 0.13] | -0.02 | [-0.14, 0.11] | 188 | -0.03 | [-0.17, 0.11] | -0.08 | [-0.24, 0.08] |
| **IL-2 (PHA, pg/mL)** | 188 | 0.07 | [-0.05, 0.18] | 0.02 | [-0.10, 0.14] | 188 | 0.07 | [-0.07, 0.22] | -0.02 | [-0.19, 0.14] |
| **IL-9 (PHA, pg/mL)** | 188 | 0.03 | [-0.09, 0.16] | -0.03 | [-0.16, 0.11] | 188 | 0.06 | [-0.09, 0.22] | -0.01 | [-0.19, 0.16] |
| **IL-10 (PHA, pg/mL)** | 188 | 0.12 | [-0.01, 0.24] | 0.06 | [-0.07, 0.20] | 188 | 0.15 | [0.00, 0.31] | 0.06 | [-0.11, 0.24] |
| **IL-13 (PHA, pg/mL)** | 188 | 0.02 | [-0.10, 0.15] | -0.03 | [-0.16, 0.10] | 188 | 0.09 | [-0.06, 0.25] | 0.03 | [-0.15, 0.20] |
| **IL-17A (PHA, pg/mL)** | 183 | 0.15 | [0.04, 0.26] | 0.10 | [-0.01, 0.22] | 183 | 0.20 | [0.06, 0.33] | 0.10 | [-0.05, 0.25] |
| **TNF-α (PHA, pg/mL)** | 188 | -0.08 | [-0.25, 0.08] | -0.05 | [-0.23, 0.13] | 188 | -0.14 | [-0.35, 0.06] | -0.09 | [-0.34, 0.15] |
| **IL-8 (basal, pg/mL)** | 193 | -0.13 | [-0.29, 0.04] | -0.05 | [-0.23, 0.12] | 193 | -0.28 | [-0.48, -0.07] | -0.18 | [-0.41, 0.06] |
| **MCP-1 (basal, pg/mL)** | 193 | 0.06 | [-0.09, 0.22] | 0.08 | [-0.09, 0.25] | 193 | -0.02 | [-0.21, 0.18] | 0.00 | [-0.23, 0.22] |
| **RANTES (basal, pg/mL)** | 193 | -0.01 | [-0.16, 0.15] | -0.02 | [-0.19, 0.15] | 193 | -0.09 | [-0.29, 0.10] | -0.14 | [-0.36, 0.09] |

*Pollutants and cytokines variables were standardized by IQR. Adjusted models were adjusted on mother age, BMI, active or passive smoking, educational level, white blood cell count, gestational age at sampling, and sampling season.*

*Abbreviations: OP: oxidative potential, AA: ascorbic acid, CI, confidence interval, IL, interleukin, IFN interferon, TNF tumor necrosis factor, RANTES regulated on activation, normal T cell expressed and secreted, MCP monocyte chemoattractant protein.*


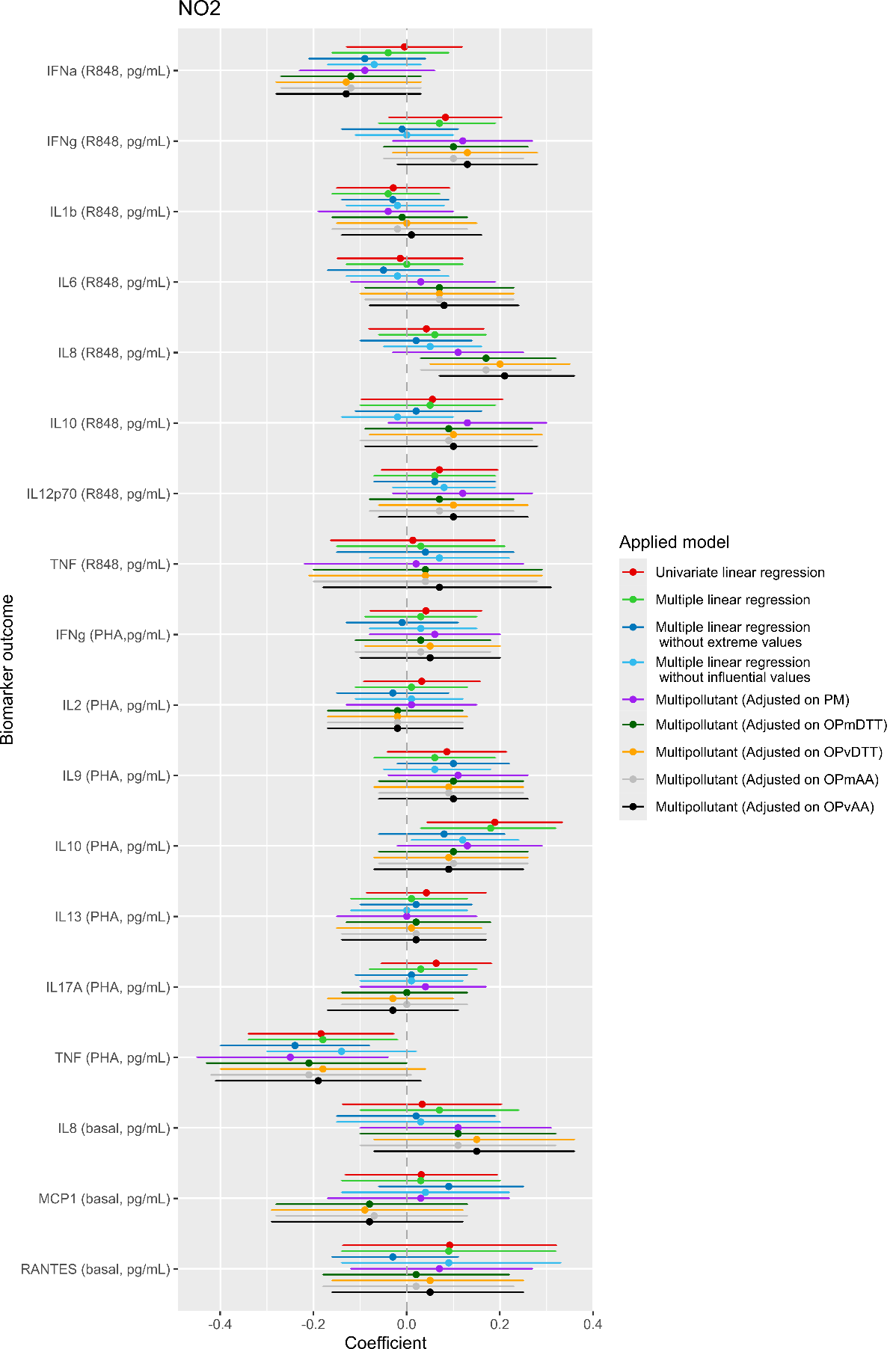


Figure S2. Sensitivity analyses of the associations between NO_2_ and cytokine levels.

Pollutants and cytokines variables were standardized by their IQR. Beta values and their 95% CI were estimated by multiple linear regression models. **Univariate**: regression model included air pollutant exposure. **Multivariate**: Regression model was adjusted for mother age, BMI, active or passive smoking, educational level, white blood cell count, gestational age at sampling, and sampling season. **Regression without extreme values:** Exclusions from the multivariate regression models were made for participants whose exposures or outcomes were outside the 1st and 99th percentiles. This exclusion accounted for approximately 2.5% of the total population. **Regression without influential values:** Multivariate regression with a Cook's distance above 4/n, where n represents the length of the regression population, were excluded from the analysis. This exclusion accounted for approximately 7% of the total population. **Multipollutant:** Multivariate regression further adjusted on another air pollutant.

*Abbreviations: NO_2_ nitrogen dioxide, CI, confidence interval, IQR, interquartile, BMI, body mass index, IL, interleukin, IFN interferon, TNF tumor necrosis factor, RANTES regulated on activation, normal T cell expressed and secreted, MCP monocyte chemoattractant protein.*


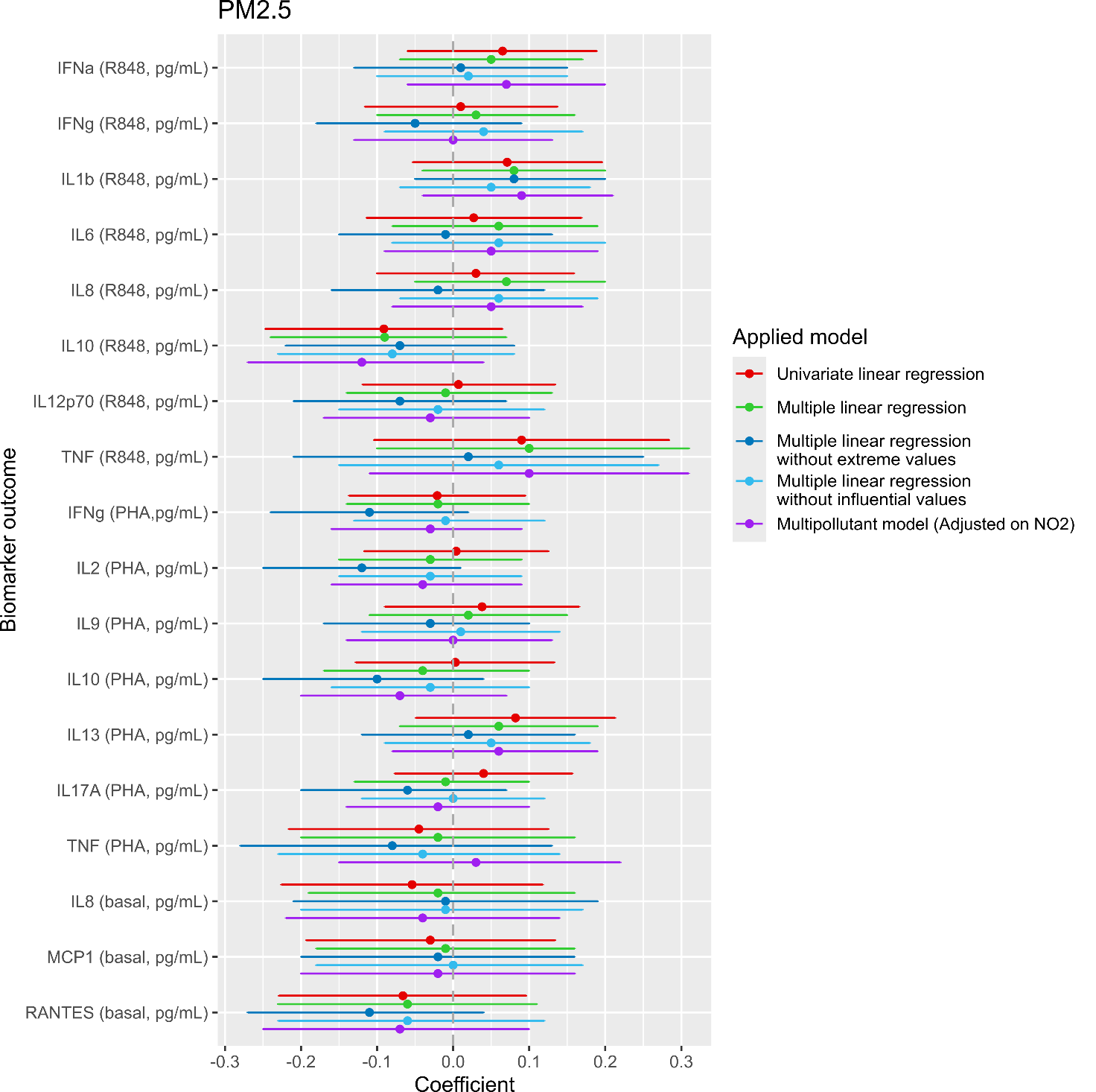


Figure S3. Sensitivity analyses of the associations between PM_2.5_ and cytokine levels.

Pollutants and cytokines variables were standardized by their IQR. Beta values and their 95% CI were estimated by multiple linear regression models. **Univariate**: regression model included air pollutant exposure. **Multivariate**: Regression model was adjusted for mother age, BMI, active or passive smoking, educational level, white blood cell count, gestational age at sampling, and sampling season. **Regression** **without extreme values**: Exclusions from the multivariate regression models were made for participants whose exposures or outcomes were outside the 1st and 99th percentiles. This exclusion accounted for approximately 2.5% of the total population. **Regression without influential values**: Multivariate regression with a Cook's distance above 4/n, where n represents the length of the regression population, were excluded from the analysis. This exclusion accounted for approximately 7% of the total population. **Multipollutant:** Multivariate regression further adjusted on another air pollutant.

*Abbreviations: PM_2.5_ particulate matter with diameter ≤ 2.5 μm, CI, confidence interval, IQR, interquartile, BMI, body mass index, IL, interleukin, IFN interferon, TNF tumor necrosis factor, RANTES regulated on activation, normal T cell expressed and secreted, MCP monocyte chemoattractant protein.*


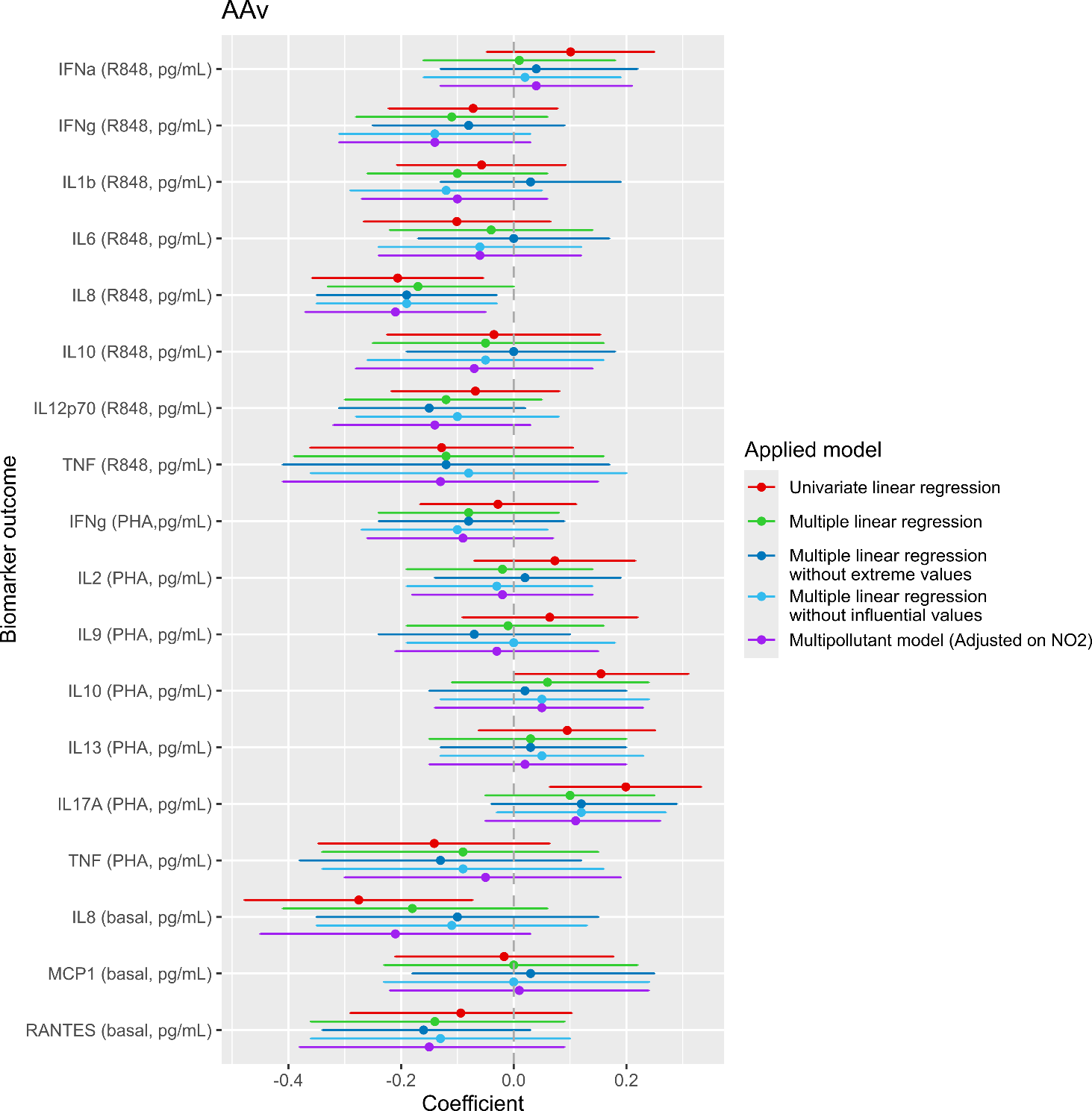


Figure S4. Sensitivity analyses of the associations between OP_v_^AA^ and cytokine levels.

Pollutants and cytokines variables were standardized by their IQR. Beta values and their 95% CI were estimated by multiple linear regression models. **Univariate**: regression model included air pollutant exposure. **Multivariate**: Regression model was adjusted for mother age, BMI, active or passive smoking, educational level, white blood cell count, gestational age at sampling, and sampling season. **Regression without extreme values**: Exclusions from the multivariate regression models were made for participants whose exposures or outcomes were outside the 1st and 99th percentiles. This exclusion accounted for approximately 2.5% of the total population. **Regression without influential values:** Multivariate regression with a Cook's distance above 4/n, where n represents the length of the regression population, were excluded from the analysis. This exclusion accounted for approximately 7% of the total population. **Multipollutant:** Multivariate regression further adjusted on another air pollutant.

*Abbreviations: OP: oxidative potential, AA: ascorbic acid, CI, confidence interval, IQR, interquartile, BMI, body mass index, IL, interleukin, IFN interferon, TNF tumor necrosis factor, RANTES regulated on activation, normal T cell expressed and secreted, MCP monocyte chemoattractant protein.*


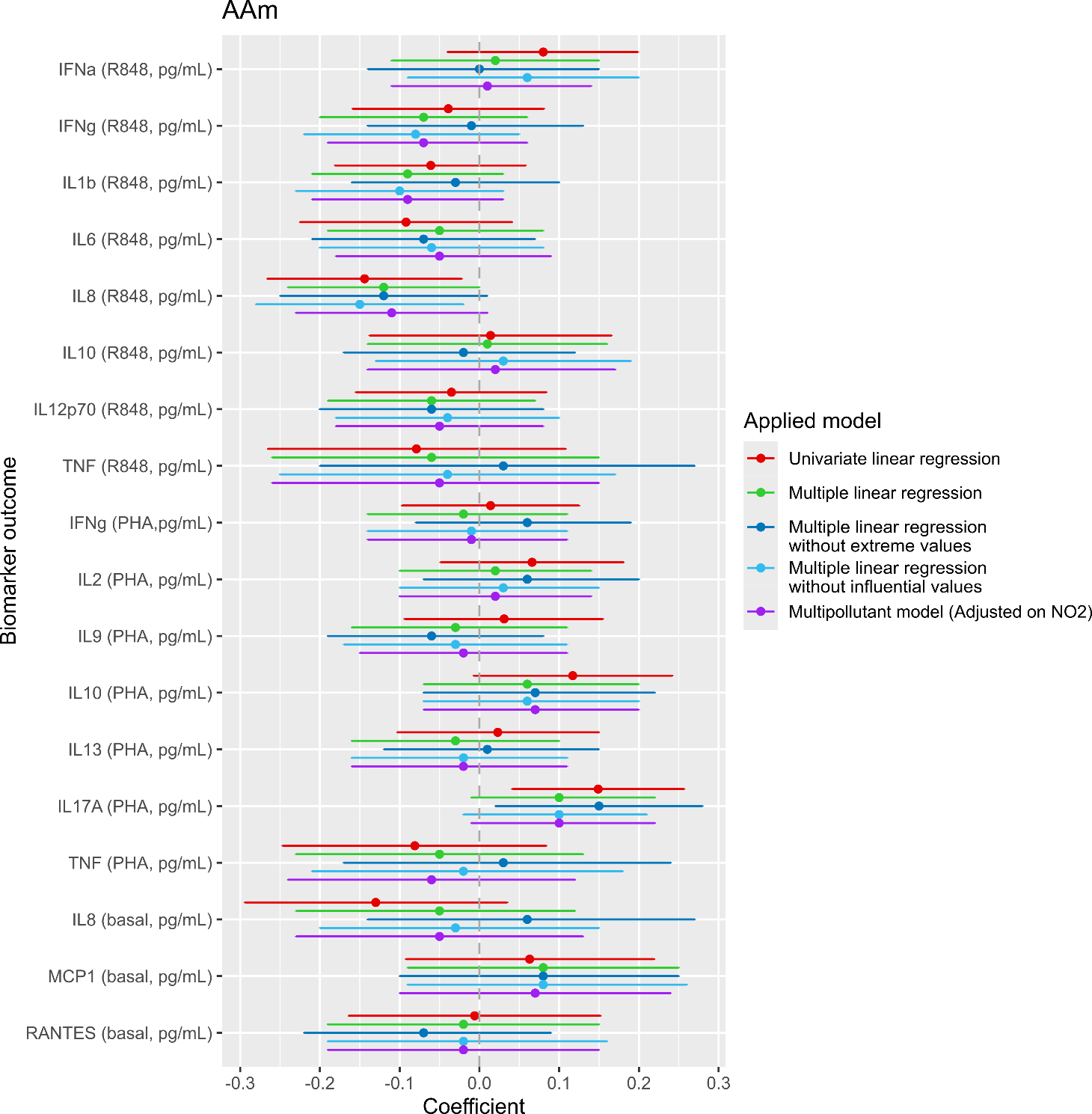


Figure S5. Sensitivity analyses of the associations between OP_m_^AA^ and cytokine levels.

Pollutants and cytokines variables were standardized by their IQR. Beta values and their 95% CI were estimated by multiple linear regression models. **Univariate**: regression model included air pollutant exposure. **Multivariate**: Regression model was adjusted for mother age, BMI, active or passive smoking, educational level, white blood cell count, gestational age at sampling, and sampling season. **Regression without extreme values:** Exclusions from the multivariate regression models were made for participants whose exposures or outcomes were outside the 1st and 99th percentiles. This exclusion accounted for approximately 2.5% of the total population. **Regression without influential values**: Multivariate regression with a Cook's distance above 4/n, where n represents the length of the regression population, were excluded from the analysis. This exclusion accounted for approximately 7% of the total population. **Multipollutant:** Multivariate regression further adjusted on another air pollutant.

*Abbreviations: OP: oxidative potential, AA: ascorbic acid, CI, confidence interval, IQR, interquartile, BMI, body mass index, Il, interleukin, IFN interferon, TNF tumor necrosis factor, RANTES regulated on activation, normal T cell expressed and secreted, MCP monocyte chemoattractant protein.*


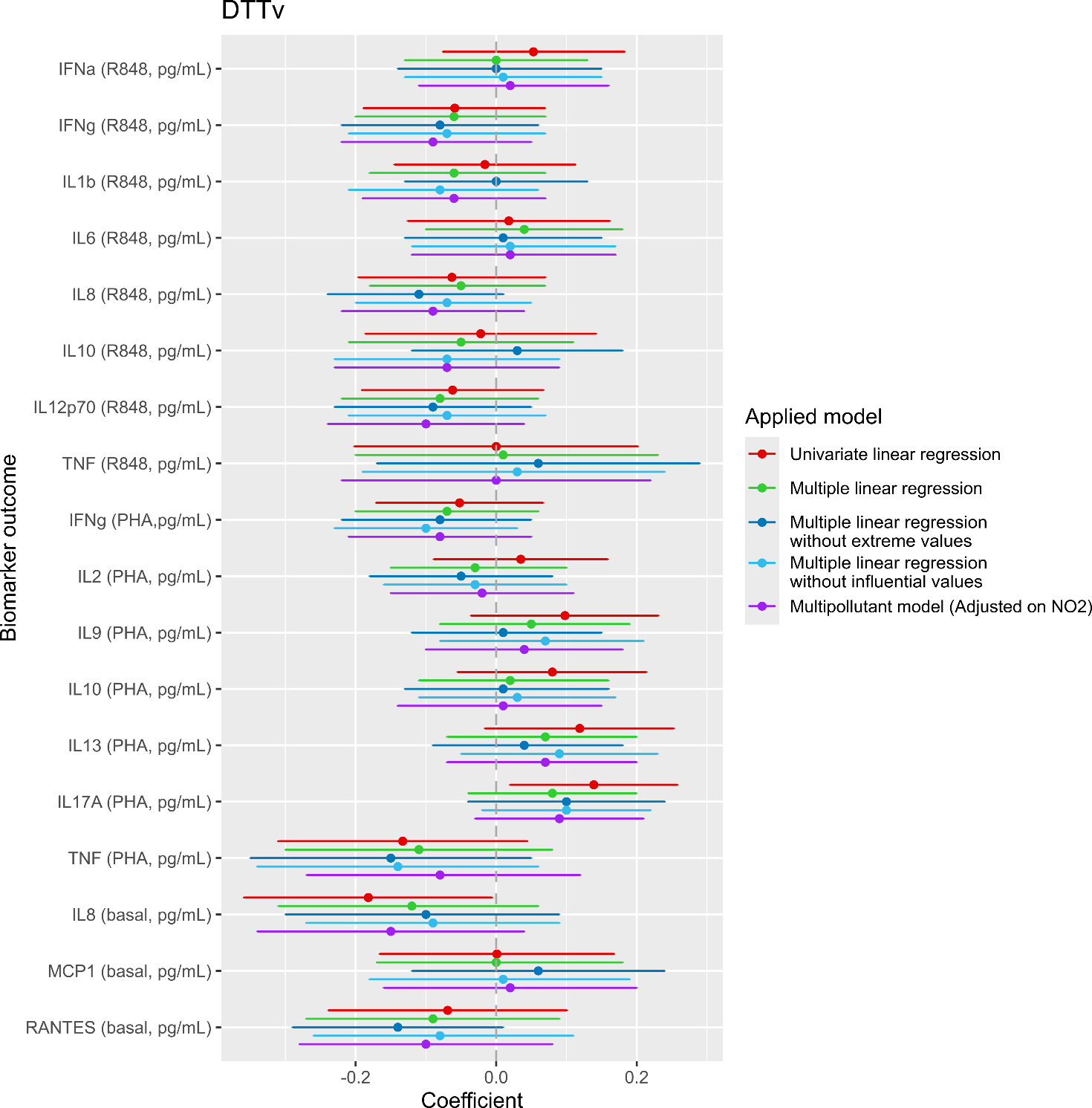


Figure S6. Sensitivity analyses of the associations between OP_v_^DTT^ and cytokine levels.

Pollutants and cytokines variables were standardized by their IQR. Beta values and their 95% CI were estimated by multiple linear regression models. **Univariate**: regression model included air pollutant exposure. **Multivariate**: Regression model was adjusted for mother age, BMI, active or passive smoking, educational level, white blood cell count, gestational age at sampling, and sampling season. **Regression without extreme values**: Exclusions from the multivariate regression models were made for participants whose exposures or outcomes were outside the 1st and 99th percentiles. This exclusion accounted for approximately 2.5% of the total population. **Regression without influential values:** Multivariate regression with a Cook's distance above 4/n, where n represents the length of the regression population, were excluded from the analysis. This exclusion accounted for approximately 7% of the total population. **Multipollutant:** Multivariate regression further adjusted on another air pollutant.

*Abbreviations: OP: oxidative potential, DTT: dithiothreitol, CI, confidence interval, IQR, interquartile, BMI, body mass index, IL, interleukin, IFN interferon, TNF tumor necrosis factor, RANTES regulated on activation, normal T cell expressed and secreted, MCP monocyte chemoattractant protein.*


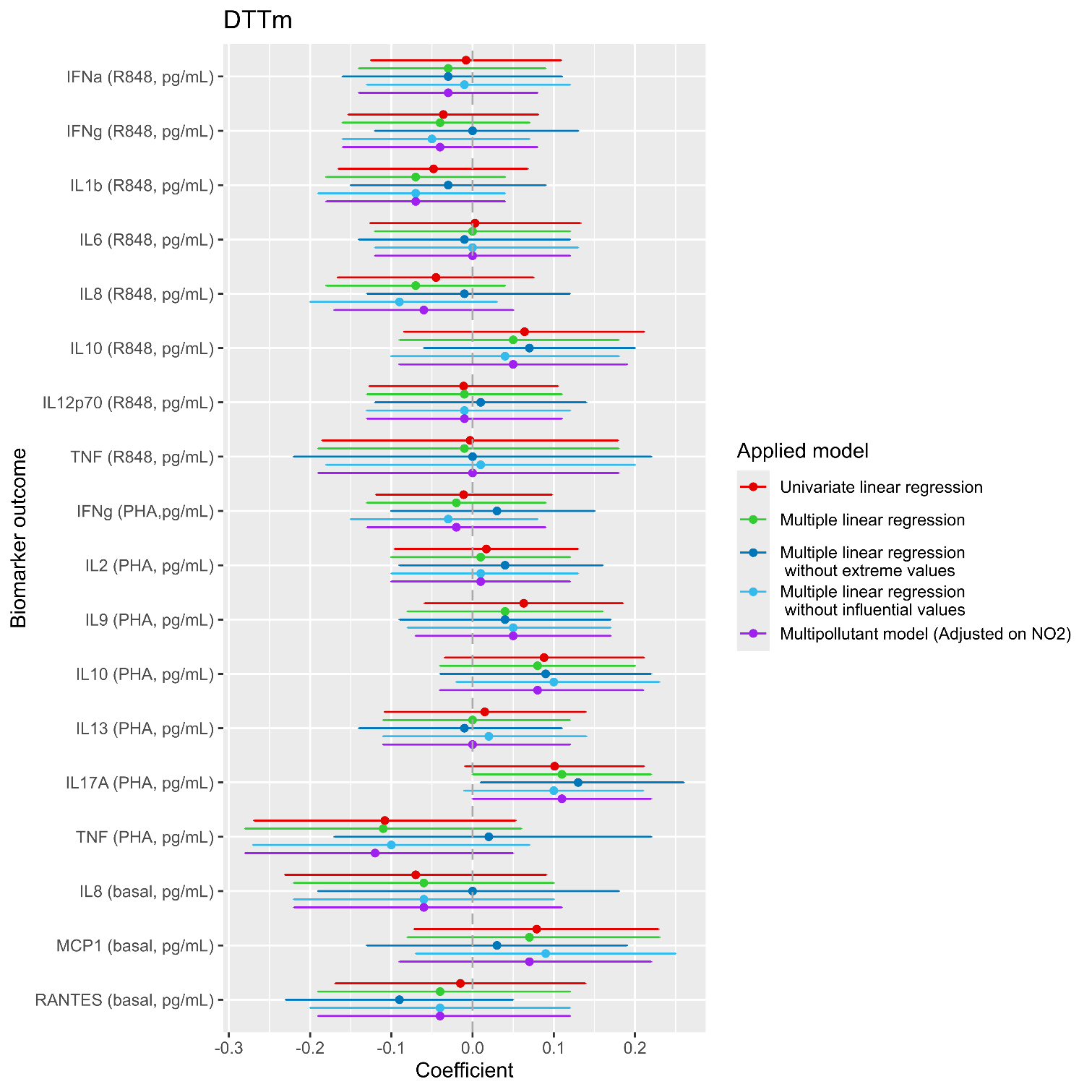


Figure S7. Sensitivity analyses of the associations between OP_m_^DTT^ and cytokine levels.

Pollutants and cytokines variables were standardized by their IQR. Beta values and their 95% CI were estimated by multiple linear regression models. **Univariate**: regression model included air pollutant exposure. **Multivariate**: Regression model was adjusted for mother age, BMI, active or passive smoking, educational level, white blood cell count, gestational age at sampling, and sampling season. **Regression without extreme values**: Exclusions from the multivariate regression models were made for participants whose exposures or outcomes were outside the 1st and 99th percentiles. This exclusion accounted for approximately 2.5% of the total population. **Regression without influential values**: Multivariate regression with a Cook's distance above 4/n, where n represents the length of the regression population, were excluded from the analysis. This exclusion accounted for approximately 7% of the total population. **Multipollutant:** Multivariate regression further adjusted on another air pollutant.

*Abbreviations: OP: oxidative potential, DTT: dithiothreitol, CI, confidence interval, IQR, interquartile, BMI, body mass index, IL, interleukin, IFN interferon, TNF tumor necrosis factor, RANTES regulated on activation, normal T cell expressed and secreted, MCP monocyte chemoattractant protein.*
